# CXCL9 inhibits tumour growth and drives anti-PD-L1 therapy in ovarian cancer

**DOI:** 10.1038/s41416-022-01763-0

**Published:** 2022-03-21

**Authors:** Stefanie Seitz, Tobias F. Dreyer, Christoph Stange, Katja Steiger, Rosalinde Bräuer, Leandra Scheutz, Gabriele Multhoff, Wilko Weichert, Marion Kiechle, Viktor Magdolen, Holger Bronger

**Affiliations:** 1 Department of Gynecology and Obstetrics, Technical University of Munich, 81675 Munich, Germany; 2 Department of Comparative Experimental Pathology, Klinikum rechts der Isar, Technical University of Munich, 81675 Munich, Germany; 3 Department of Radiation Oncology, Technical University of Munich, TranslaTUM, 81675 Munich, Germany; 4 Department of Pathology, Technical University of Munich, 81675 Munich, Germany; 5 German Cancer Consortium (DKTK), partner site Munich, and German Cancer Research Center (DKFZ), Heidelberg, Germany

**Keywords:** Ovarian cancer, Tumour immunology, Chemokines, Immunotherapy

## Abstract

**Background:**

Response to immune checkpoint blockade (ICB) in ovarian cancer remains disappointing. Several studies have identified the chemokine CXCL9 as a robust prognosticator of improved survival in ovarian cancer and a characteristic of the immunoreactive subtype, which predicts ICB response. However, the function of CXCL9 in ovarian cancer has been poorly studied.

**Methods:**

Impact of Cxcl9 overexpression in the murine ID8-*Trp53*^*−/−*^ and ID8-*Trp53*^*−/–*^*Brca2*^*−/−*^ ovarian cancer models on survival, cellular immune composition, PD-L1 expression and anti-PD-L1 therapy. CXCL9 expression analysis in ovarian cancer subtypes and correlation to reported ICB response.

**Results:**

CXCL9 overexpression resulted in T-cell accumulation, delayed ascites formation and improved survival, which was dependent on adaptive immune function. In the ICB-resistant mouse model, the chemokine was sufficient to enable a successful anti-PD-L1 therapy. In contrast, these effects were abrogated in *Brca2*-deficient tumours, most likely due to an already high intrinsic chemokine expression. Finally, in ovarian cancer patients, the clear-cell subtype, known to respond best to ICB, displayed a significantly higher proportion of CXCL9^high^ tumours than the other subtypes.

**Conclusions:**

CXCL9 is a driver of successful ICB in preclinical ovarian cancer. Besides being a feasible predictive biomarker, CXCL9-inducing agents thus represent attractive combination partners to improve ICB in this cancer entity.

## Background

Despite innumerable efforts to improve outcome, ovarian cancer still is one of the deadliest cancers in women worldwide. First-line therapy usually comprises radical upfront debulking surgery, followed by adjuvant, platinum-based chemotherapy. In recent years, inhibitors of the poly (ADP-ribose) polymerase (PARPi) have successfully complemented this armamentarium, especially in tumours exhibiting a homologous recombination repair deficiency such as a BRCA1/2 mutation [1]. Immuno-oncology, most notable inhibition of the PD-1/PD-L1 checkpoint (ICB), which has revolutionised the therapy of several other tumour entities, has not yet shown activity in ovarian cancer with response rates remaining disappointingly low [2]. Causative might be a comparatively low tumour mutational burden (TMB), an immune-suppressive tumour microenvironment enriched with regulatory T cells and tumour-associated macrophages, and limited access of the tumour microenvironment for T-effector cells [3]. Tumour-infiltrating lymphocytes (TILs) represent a strong and robust prognostic marker for improved outcome in ovarian cancer, suggesting that an effective anti-tumour response is, in principle, possible [4–6].

The chemokine CXCL9, along with its two family members CXCL10 and CXCL11, promotes tumour-suppressive lymphocytic infiltration in solid tumours via its receptor CXCR3 [7]. In addition, CXCR3 chemokine action has been demonstrated to be vital for successful immune checkpoint inhibition in preclinical cancer models, due to both T-cell recruitment and activation [8–10]. Consistently, a recent report identified CXCL9 gene expression as the most powerful marker besides TMB to predict ICB response in >1000 patients across multiple tumour types [11].

In ovarian cancer, CXCL9 overexpression has been shown to correlate with enhanced T-cell infiltration and improved overall survival [4, 12, 13]. Several unsupervised studies, aiming to define the molecular subtypes of ovarian cancer by gene expression profiling, univocally identified CXCL9 and the other CXCR3 chemokines as marker genes of an inflammation-enriched subtype [14–18]. In a subsequent meta-analysis, combining 14 of these studies including the TCGA datasets and looking at more than 900 genes, the CXCR3 chemokines were the three most upregulated genes in the so-called “immunoreactive subtype” [19]. This subtype showed the best response to ICB treatment in ovarian cancer patients [20]. Moreover, in an attempt to decipher genes associated with tumour regression and therapy response, CXCL9 emerged once again as the most upregulated gene in regressive, chemosensitive metastases of ovarian cancer patients [21]. Despite this considerable evidence indicative of a crucial role of CXCL9 in ovarian cancer biology, its actual function therein has been poorly investigated.

In this study, we asked if CXCL9 also functionally fulfils the protective role suggested by the correlative studies above. In addition, given its dominance in the immunoreactive subtype, we wanted to know if its expression is sufficient to enable efficient ICB in ovarian cancer. Our study provides evidence for a central role of this chemokine in ovarian cancer immune intervention and renders CXCL9 a promising predictor and driver of ICB response in this cancer entity.

## Materials and methods

### Human tissue samples and patient characteristics

For immunohistochemical studies, ovarian cancer patients who underwent surgery between 1990 and 2014 at the Technical University of Munich Hospital (Klinikum Rechts der Isar, Department of Obstetrics and Gynecology) were included in our study. A pathologist reviewed all samples to confirm histological subtypes: clear-cell carcinoma (*n* = 16), mucinous carcinoma (*n* = 16), borderline carcinoma (*n* = 18), endometrioid carcinoma (*n* = 30) and low-grade serous carcinoma (*n* = 15). In addition, we included 178 high-grade serous carcinoma cases that were already described by us before [12]. Written informed consent was obtained from all patients.

### Immunohistochemistry on human samples

CXCL9 immunohistochemistry was performed on Tissue Microarrays (TMAs) with formalin-fixed, paraffin-embedded (FFPE) tumour cores from each patient in triplicates. Briefly, 3-µm sections were deparaffinized by treatment with xylene followed by a graded series of ethanol and rehydrated in distilled water. Heat-induced epitope retrieval was performed by pressure cooking in citrate buffer (pH 6). Sections were washed thoroughly with TBS-T between incubations. Endogenous peroxidase activity was quenched by 20 min (RT) incubation with 3% H_2_O_2_ and subsequent wash with tap water, followed by 10 min (RT) antigen blocking with 5% goat serum. Primary polyclonal rabbit CXCL9 antibody (Cat. #PA5-34743, Invitrogen) was diluted 1:100 in antibody diluent (ZUC025, Zytomed Systems) and incubated for 1 h at room temperature. For detection of primary antibody binding, Zyto-Chem Plus HRP One-Step Polymer anti-mouse/rabbit (Cat. #ZUC053, Zytomed Systems) and DAB Substrate Kit (DAB530, Zytomed) were applied according to the manufacturer’s instructions. Sections were counterstained with hematoxylin and blued under tap water. Dehydration with an ascending alcohol series and mounting with Pertex was performed afterwards. Histological images were taken using the digital slide scanner NanoZoomer Digital Pathology RS (Hamamatsu, Japan) and semiquantitatively analysed with NDP.view 2 software version 2.8.24.

### Cell lines and cell culture

The ID8-*Trp53*^−*/−*^ and ID8-*Trp53*^*−/−*^*Brca2*^−*/−*^ mouse ovarian surface epithelial cells were kindly provided by Prof. Iain McNeish’s laboratory (University of Glasgow) [22]. The human packaging cell line HEK293T was obtained from DSMZ (Leibniz Institute). Both cell lines were cultured in Dulbecco’s modified Eagle’s medium (DMEM) supplemented with 10% foetal calf serum (FCS) and 10 mM HEPES. ID8 derivates additionally received 1% ITS solution containing 10 µg/mL insulin, 5.5 µg/mL transferrin and 6.7 ng/mL sodium selenite. Human ovarian cancer cell lines CAOV-3, OVCAR-3 and SKOV-3 were purchased from the American Type Culture Collection (ATCC) [23]. OV-MZ-6 cells were obtained from Möbus et al. [24]. CAOV-3 and OV-MZ-6 cells were cultured in DMEM (10% FCS, 10 mM HEPES), OVCAR-3 cells in RPMI 1640 media (20% FCS, 0.1 µg/mL bovine pancreas insulin solution, 10 mM HEPES) and SKOV-3 cells in McCoy’s 5 A media (10% FCS, 10 mM HEPES). Cell lines were grown in a humidified 5% CO_2_ atmosphere at 37 °C, gently harvested with 0.05% EDTA or 0.25% trypsin solution and regularly tested for mycoplasma contamination.

### Stable overexpression of murine Cxcl9

Overexpression of murine Cxcl9 in ID8-*Trp53*^*−/−*^ and ID8-*Trp53*^*−/−*^*Brca2*^−*/−*^ cells was conducted with the ViraSafe^TM^ Lentiviral Expression System (Neo) from Cell Biolabs (Cat. #VPK-213-ECO). cDNA derived from Cxcl9 mouse untagged clone Cat. #MC200015 (OriGene Technologies) was ligated into the multiple cloning site of the pSMPUW-Neo transfer vector. Selection of cell clones for incorporated pSMPUW-Neo was achieved by G418 (1 mg/mL) addition to the cell media. After 1 week, single-cell clones were selected by limiting dilution. As a control, target cells were treated analogously with the pSMPUW-Neo-empty vector. Validation of Cxcl9 overexpression was performed applying the mouse Cxcl9/MIG DuoSet ELISA Kit (Cat. #DY492) from R&D Systems in the supernatant of cultured cells as well as via qPCR.

### Animal experiments

Approval for all experimental animal procedures was obtained from the Government of Upper Bavaria (Regierung von Oberbayern). Projects were carried out in accordance with the institutional guidelines of the Preclinical Research Center at the Technical University of Munich. Cell lines underwent mycoplasma testing before in vivo application. Key experiments were performed at least twice. Six- to eight-week-old female C57BL/6 (strain 632) and athymic nude mice (strain 490) were obtained from Charles River. To evaluate the effect of intratumoral Cxcl9 on survival, ovarian cancer was induced by intraperitoneal (IP) administration of 1 × 10^7^ ID8-*Trp53*^*−/−*^*Cxcl9*^*+*^ or ID8-*Trp53*^*−/−*^*Brca2*^−*/−*^*Cxcl9*^*+*^ cells dissolved in 250 µl of phosphate-buffered saline (PBS). Control groups were inoculated with corresponding empty vector clones. In vivo experiments were performed with four to seven mice per group. Tumour cell injection resulted in diffuse carcinomatosis throughout the abdominal cavity and haemorrhagic ascites formation. Visually recognisable abdominal swelling was defined as the onset of ascites. Animals were euthanized upon reaching predefined endpoints (severe accumulation of ascitic fluid and/or poor health condition). Ascitic fluid was collected from the peritoneal cavity for soluble chemokine analysis. Mesentery tumour implants were collected, fixed in 4% paraformaldehyde and embedded in paraffin for immunohistochemical staining.

Mice were randomised after tumour cell inoculation to either receive anti-PD-L1 or isotype control therapy. Therapy started 30 days after tumour cell inoculation via intraperitoneal injection of 200 µg *InVivo*MAb anti-mouse PD-L1 (B7-H1) Clone: 10.F.9G2 (Cat. #BE0101, Bio X Cell) or *InVivo*MAb rat IgG2b isotype control Clone: LTF-2 (Cat. #BE0090, Bio X Cell). Antibodies were diluted in *InVivo*Pure pH 7.0 Dilution Buffer (Cat. #IP0070, Bio X Cell) and injected at 2 µg/µl dosage twice a week until endpoints were reached.

### Evaluation of ascitic chemokine concentrations with ELISA

Ascitic fluid was centrifugated and the supernatant was used in Cxcl9 and Cxcl10 DuoSet ELISA Kits from R&D Systems (Cat. #DY492 and #DY466). The resulting chemokine concentrations were normalised to total protein amounts of ascitic supernatants, determined by a Bradford assay with Coomassie Brilliant Blue G-250 (Cat. #27815, Sigma-Aldrich) and bovine serum albumin (Cat. #1470, Sigma-Aldrich).

### Immunohistochemistry on murine tumour tissue

Formalin-fixed, paraffin-embedded mesentery tumour tissue was cut into 3-µm sections. Ki-67, F4/80 and DX5 stainings were performed using the Zyto-Chem Plus HRP One-Step Polymer System as well as epitope retrieval and peroxide block as mentioned above. The following dilutions of primary antibodies were applied: 1:1000 for the polyclonal rabbit Ki-67/MKI67 antibody (Cat. #NB500-170, Novus Biologicals); 1:300 for the monoclonal rabbit F4/80 (D2S9R) XP^®^ antibody (Cat. #70076, Cell Signaling Technology), and 1:50 for the monoclonal rat CD49b/DX5 antibody (Cat. #108902, BioLegend).

Additional stainings were performed on an automated immunostainer (Agilent Technologies) including heat-induced epitope retrieval with citrate buffer pH 6.0 or EDTA buffer pH 9.0 and using the following primary antibody dilutions: 1:100 for the rabbit CD3 antibody (clone SP7, Cat. #CI597C01, *DCS* Diagnostics); 1:100 for the monoclonal rat CD8 antibody (clone GHH8, Cat. #DIA-808, Dianova); 1:2000 for the polyclonal rabbit Foxp3 antibody (Cat. #ab4728, Abcam); 1:50 for the monoclonal mouse PD-1 (NAT105) antibody (Cat. #315, Cell Marque) and 1:1000 for the polyclonal rabbit granzyme B antibody (Cat. #ab4059, Abcam). Signal detection was achieved applying the BOND Polymer Refine Detection Kit (Cat. #DS9800, Leica Biosystems) including peroxide block, post-primary reagent, DAB chromogen and haematoxylin for counterstaining.

Stained slides were scanned using the slide scanner AT-2 (Leica Biosystems) and representative images were taken at 40-fold magnification in Aperio ImageScope software version 12.3 (Leica Biosystems). QuPath open-source software version 0.2.3 [25] was used to evaluate DAB-positive cells in five intratumoral regions of interest (ROIs) with a mean cell detection of 2000 cells each.

### In silico analysis

Publicly available data of “The Cancer Genome Atlas” (TCGA) project was used at the cBioPortal website to correlate *CXCL9* and *PD-L1* mRNA expression among 489 patients with ovarian serous cystadenocarcinoma. In addition, publicly available data from the cBioPortal website was used to compare *CXCL9* mRNA expression among 47 human ovarian carcinoma cell lines, including eight distinct histological subtypes.

### Statistics

Statistical analysis was performed using GraphPad Prism Software version 9.0.1 (GraphPad Software). In vitro experiments were conducted at least three times. Bars and horizontal lines represent the mean ± standard error of the mean (SEM). Each dot represents an individual mouse. Significant differences between groups were evaluated using ANOVA. Frequency counts were compared with a two-tailed Fisher’s exact test. Correlations are shown with Pearson correlation coefficients. Kaplan–Meier estimates of event-free survival were compared by log-rank tests. *P* values <0.05 were considered significant.

Additional methods are described in the Supplementary Materials and Methods.

## Results

### CXCL9 overexpression prolongs survival through activation of the adaptive immune system in the syngeneic ID8-*Trp53*^*−/−*^ ovarian cancer mouse model

**Fig. 1:**
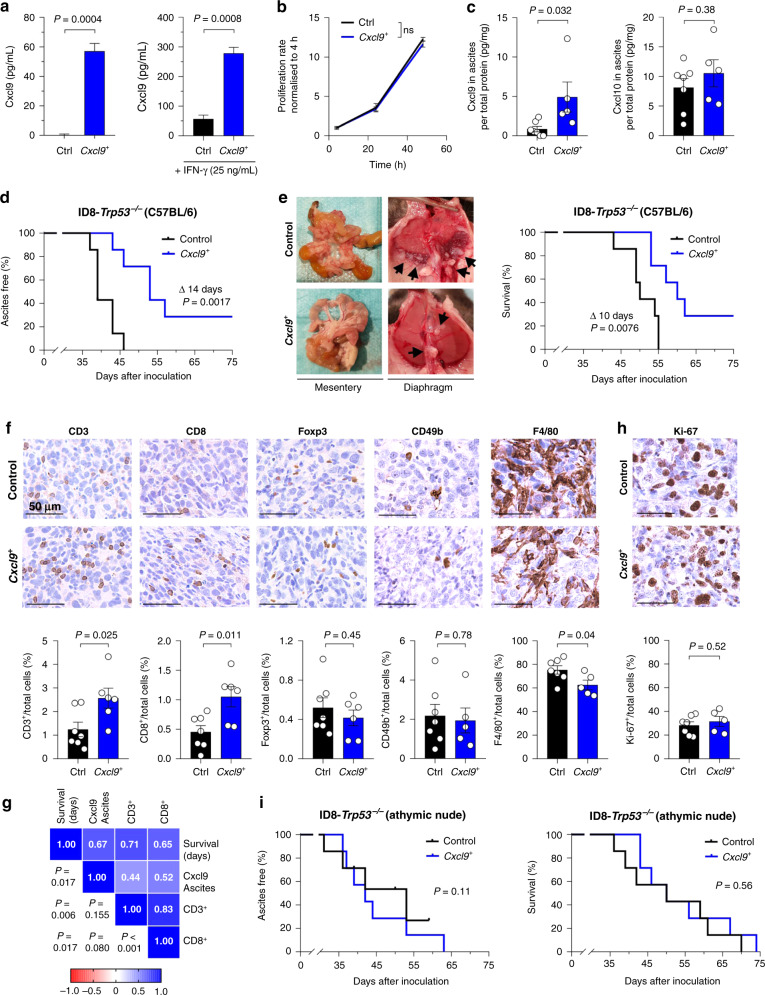
Overexpression of Cxcl9 significantly improves survival in vivo, dependent on the adaptive immune system. **a** Soluble Cxcl9 was measured in cell supernatants of ID8-*Trp53*^*-/-*^Control (Ctrl) and ID8-*Trp53*^−*/−*^*Cxcl9*^*+*^ (*Cxcl9*^*+*^) cells after 48 h  ± IFN-γ (25 ng/mL) stimulation by ELISA (*n* = 3). **b** Proliferation rates of ID8-*Trp53*^−*/−*^Control and *Cxcl9*^*+*^ cells were measured via MTT assay and normalised to the baseline value at 4 h (*n* = 3). **c**–**g** In all, 1 × 10^7^ ID8-*Trp53*^−*/−*^ Control or *Cxcl9*^*+*^ cells were intraperitoneally inoculated into C57BL/6 mice. **c** Soluble Cxcl9 or Cxcl10 in murine ascites was determined in ID8-*Trp53*^*−/−*^ Control and *Cxcl9*^*+*^ groups by ELISA and normalised to total protein assessed by Bradford assay. **d** Kaplan–Meier plot showing the time to onset of ascites of animals inoculated with ID8-*Trp53*^*−/−*^Control or *Cxcl9*^*+*^ tumour cells (data representative of two independent experiments). **e** Representative pictures of reduced diaphragmatic and mesenteric tumour load in ID8-*Cxcl9*^*+*^ compared to control tumour-bearing mice finalised on the same day. Adjacent Kaplan–Meier plot (right) shows overall survival of animals inoculated with ID8-*Trp53*^*−/−*^Control or *Cxcl9*^*+*^ tumour cells (data representative of two independent experiments). **f** Representative pictures of immunohistochemically stained markers (CD3, CD8, Foxp3, CD49b, F4/80) of intratumoral immune cell populations in ID8-*Trp53*^*−/−*^Control and *Cxcl9*^*+*^ tissue sections (scale bars 50 µm) with corresponding column diagrams of digitally analysed tissue sections below. **g** Heatmap containing Pearson correlation coefficients and associated significances between survival, ascites Cxcl9 and intratumoral CD3^+^ or CD8^+^ relative cell numbers of ID8-*Trp53*^−*/−*^ tumours. **h** Digital analysis of IHC Ki-67-positive proliferating cells within the tumour microenvironment. **i** In total, 1 × 10^7^ ID8-*Trp53*^*−/−*^Control or *Cxcl9*^*+*^ cells were intraperitoneally inoculated into athymic nude mice. Kaplan–Meier plots showing time to onset of ascites (left) or time to death (right). Bars represent mean ± SEM. Each dot indicates data of one individual mouse. Mouse experiments were conducted with seven mice per group.

To study the functional role of CXCL9 in ovarian cancer, we first engineered murine ovarian cancer ID8-*Trp53*^−*/−*^ cells to stably overexpress murine CXCL9 (Cxcl9) via lentiviral transduction. Hereinafter, these ID8-*Trp53*^*−/−*^*Cxcl9*^*+*^ and ID8-*Trp53*^−*/−*^*empty vector* cells will be referred to as “*Cxcl9*^*+*^” and “Control” cells, respectively. Under the cell culture conditions chosen (12-well plates, 80% confluency, 48 h, 500 µl volume of cell supernatant), *Cxcl9*^*+*^ cells secreted 57 pg/mL, whereas no Cxcl9 was detectable in the supernatants of Control cells (*P* = 0.0004; Fig. 1a), reflecting differentially CXCL9-expressing ovarian cancer cells (Supplementary Fig. S1A) or cancer microenvironments [12]. Similar findings were made at the mRNA level (Supplementary Fig. S1B). When cells were stimulated with IFN-γ (25 ng/mL), one of the most potent inducers of CXCR3 chemokines in vitro and in vivo, *Cxcl9*^*+*^ cells still secreted about fivefold more Cxcl9 than Control cells (279 vs. 57 pg/mL; *P* = 0.0008; Fig. 1a). Cxcl9 overexpression did not impact cell proliferation in vitro (Fig. 1b). Next, 1 × 10^7^ *Cxcl9*^*+*^ or Control cells were implanted intraperitoneally into immunocompetent C57BL/6 mice. In vivo, overexpression of Cxcl9 was preserved in about the same range as observed in vitro, as detected in the ascites of tumour-bearing mice at the end of the experiment (median 3.1 vs. 0.5 pg/mg total protein; *P* = 0.032; Fig. 1c). Cxcl10 ascites concentrations, by contrast, were not affected by *Cxcl9* tumour cell overexpression (median 11.0 vs. 9.8 pg/mg total protein; *P* = 0.38; Fig. 1c), suggesting that expression levels of CXCR3 chemokines in the ascites of ovarian cancer patients are mainly driven by tumour cell expression as proposed before by our group [12, 26].

Onset of ascites, indicated by visible abdominal swelling and one of the cardinal symptoms in ovarian cancer patients, was significantly delayed in *Cxcl9*^*+*^ tumour-bearing hosts compared to the Control group (median 53 vs. 39 days; *P* = 0.002; Fig. 1d). More importantly, Cxcl9 overexpression led to decreased peritoneal metastasis (e.g. to the diaphragm or the mesentery) and significantly prolonged median survival of the mice (60 vs. 50 days; *P* = 0.008; Fig. 1e).

As CXCR3 chemokines are thought to contribute to tumour-suppressive lymphocytic infiltration, we next quantified different intratumoral immune cell subsets by digital analysis of immunohistochemically stained mesenteric tumour tissues (Fig. 1f). The percentage of intratumoral CD3^+^ cells was augmented 2.7-fold in the *Cxcl9*^*+*^ group compared to Control tissue (median 2.55% vs. 0.96% of total cells; *P* = 0.025). Similarly, the number of CD8^+^ cells was 2.5 times higher upon Cxcl9 overexpression (median 1.19% vs. 0.47% of total cells; *P* = 0.011). There was no significant difference between both groups regarding the recruitment of Foxp3^+^ regulatory T cells (median 0.47% vs. 0.42%; *P* = 0.45) or CD49b^+^ natural killer cells (median 1.61% vs. 1.87%; *P* = 0.78). However, we noticed a small but significant decrease of F4/80-positive tumour-associated macrophages in *Cxcl9*^*+*^ tumours (median 60.2% vs. 74.5%; *P* = 0.04). Ascites concentration of Cxcl9, as well as the number of intratumoral CD3^+^ and CD8^+^ T cells, were significantly associated with improved outcome in the ID8-*Trp53*^*−/−*^ model (Fig. 1g). Moreover, there was a moderate correlation between Cxcl9 ascites concentration and the number of tumour-infiltrating T cells (Fig. 1g).

Next, we asked what mechanism drives Cxcl9-mediated tumour suppression in ovarian cancer. Ki-67 positivity was identical in *Cxcl9*^*+*^ and Control tumours (median 32.6% vs. 30.6%; *P* = 0.52; Fig. 1h), confirming our in vitro results (Fig. 1b), thus excluding changes in cell proliferation by Cxcl9. To test to what extent the improved survival of mice with implanted ID8-*Trp53*^−*/−*^*Cxcl9*^*+*^ tumours is dependent on the adaptive immune system as suggested by the increase in T-cell infiltration, we repeated the experiments in immunodeficient athymic nude mice. Here, the Cxcl9-induced survival benefit was completely abrogated both regarding time to onset of ascites (median *Cxcl9*^*+*^ 42 days vs. Control 53 days; *P* = 0.11) and survival time (median 50 days in both groups; *P* = 0.56; Fig. 1i), although Cxcl9 ascites concentration was again significantly higher in the *Cxcl9*^*+*^ group (median 2.1 vs. 0.1 pg/mg total protein; *P* = 0.013; Supplementary Fig. S1C). As expected, there was no change in the number of tumour-infiltrating T cells between both groups in athymic mice (Supplementary Fig. S1D). Thus, activation of the adaptive immune response seems to be the major mechanism behind the protective nature of CXCL9 in ovarian cancer, possibly more important than chemotactic retention of CXCR3-positive tumour cells in the primary tumour as proposed before [26].

### CXCL9 overexpression enables successful anti-PD-L1 immune checkpoint inhibition in the ID8-*Trp53*^*−/−*^ ovarian cancer model

**Fig. 2:**
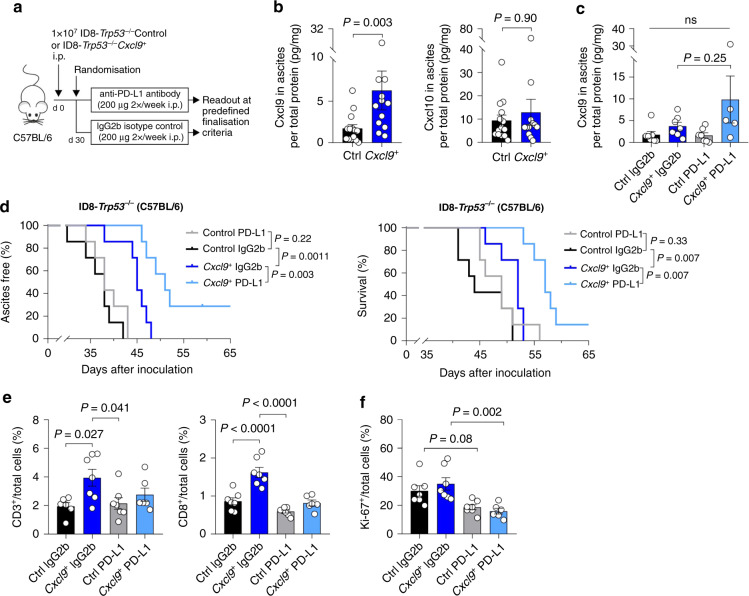
Cxcl9 overexpression enables functional immune checkpoint blockade in ID8-*Trp53*^*−/*−^ tumour-bearing mice. **a** Experimental setup used in (**b**–**f**): C57BL/6 mice were intraperitoneally injected with 1 × 10^7^ ID8-*Trp53*^*−/−*^Control (Ctrl) or ID8-*Trp53*^*−/−*^*Cxcl9*^*+*^ (*Cxcl9*^*+*^) cells and treated with 200 µg anti-PD-L1 antibody or corresponding IgG2b isotype control. **b**, **c** Soluble Cxcl9 or Cxcl10 in murine ascites of groups listed under section (**d**) was quantified by ELISA and normalised to total protein. **d** Kaplan–Meier plots with time to ascites formation (left) and overall survival (right) of anti-PD-L1 or IgG2b-treated groups stratified by Cxcl9 overexpression in the ID8-*Trp53*^*−/−*^ model (*n* = 7 in each group). **e**, **f** Digital analysis of immunohistochemically detected immune cell population markers (CD3, CD8, Foxp3) and Ki-67-positive tumour cells within the tumour microenvironment. Each dot indicates the data of one individual mouse. Bars represent mean ± SEM.

As ICB monotherapy still does not satisfactorily work in ovarian cancer patients [27], and as the CXCR3 chemokine system has been reported to take part in therapy response to ICB in preclinical cancer models [9, 13], we asked whether CXCL9 overexpression all by itself was sufficient to facilitate a successful anti-PD-L1 therapy. To this end, C57BL/6 mice harbouring ID8-*Trp53*^*−/−*^*Cxcl9*^*+*^ or Control tumours were randomised to receive either anti-PD-L1 antibody or an IgG2b isotype control antibody twice a week (Fig. 2a). Cxcl9 was about threefold higher expressed in ascites in *Cxcl9*^*+*^ tumour-bearing mice than in the control group (median 4.58 vs. 1.45 pg/mg total protein; *P* = 0.003; Fig. 2b), whereas there was no significant change in Cxcl10 (median 6.62 vs. 6.07 pg/mg total protein; *P* = 0.90; Fig. 2b). In contrast to prior reports demonstrating induction of CXCR3 chemokines upon immune checkpoint inhibition in preclinical models other than ovarian cancer [9], we did not observe such an increase in the ID8 ovarian cancer model (Fig. 2c and Supplementary Fig. S2A). In Control tumours, anti-PD-L1 therapy did not affect the time to onset of ascites or survival (Fig. 2d), thus accurately reflecting the situation in human ovarian cancer. However, anti-PD-L1 treatment acted synergistically with Cxcl9 overexpression and significantly prolonged time to onset of ascites (median 51 vs. 45 days; *P* = 0.003) and overall survival (median 57 vs. 52 days; *P* = 0.007) in the *Cxcl9*^*+*^ tumour group (Fig. 2d).

Next, we looked at immune infiltration. Unexpectedly, despite an improved ICB treatment, we did not observe an increase in the CD3^+^ or CD8^+^ immune infiltration upon Cxcl9 overexpression in the tumours treated with the anti-PD-L1 antibody, which we once again reproduced in the IgG2b-treated tumours (Fig. 2e). Foxp3^+^ regulatory T cells and F4/80^+^ macrophages remained unchanged (Supplementary Fig. S2B). A significant reduction in cell proliferation as determined by Ki-67 positivity was induced by ICB treatment in *Cxcl9*^*+*^ tumours (median 15.3% vs. 30.3%; *P* = 0.002; Fig. 2f). There was no change in granzyme B or PD-1-positive immune cells (Supplementary Fig. S2C).

### Cxcl9-induced survival benefit and ICB effectiveness diminish in *Brca2*-deficient tumours

**Fig. 3:**
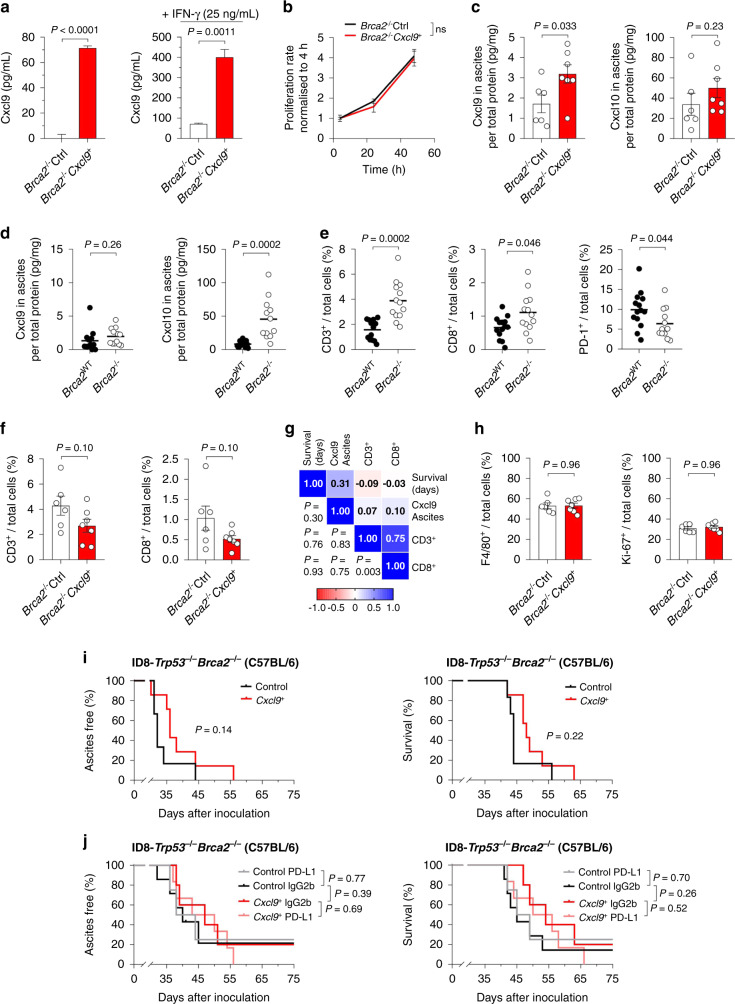
Cxcl9 loses its ability to suppress tumour growth and to provide ICB responsiveness in a *Brca2-*deficient mouse model. **a** Cxcl9 overexpression was assessed in ID8-*Trp53*^*−/−*^*Brca2*^−*/−*^Control (*Brca2*^*−/*−^Ctrl) and ID8-*Trp53*^*−/−*^*Brca2*^*−/−*^*Cxcl9*^*+*^ (*Brca2*^*−/−*^*Cxcl9*^*+*^) cell supernatants after 48 h stimulation with solvent control (left) or 25 ng/mL IFN-γ (right) by ELISA (*n* = 3). **b** Proliferation of *Brca2*^*−/−*^Ctrl and *Brca2*^*−/−*^*Cxcl9*^*+*^ cells was assessed via MTT assay and normalised to 4 h baseline values (*n* = 3). **c** Cxcl9 and Cxcl10 concentrations relative to total protein in ascites of ID8-*Trp53*^*−/−*^*Brca2*^−*/−*^Control or *Cxcl9*^*+*^ tumour-bearing mice were calculated via ELISA. **d** Impact of *Brca2* loss-of-function on relative Cxcl9 and Cxcl10 ascites concentrations as determined by ELISA of ascites from tumour-bearing mice. **e** Number of CD3^+^, CD8^+^ and PD-1^+^ immune cells in wild-type and *Brca2*-deficient tumours as determined by digital analysis of immunohistochemical stainings. **f** Relative abundance of CD3^+^ and CD8^+^ T cells in ID8-*Trp53*^*−/−*^*Brca2*^*−/−*^Control and *Cxcl9*^*+*^ tumours. **g** Heatmap correlation matrix showing Pearson correlation coefficients and associated significances between survival, ascites Cxcl9 concentration and intratumoral CD3^+^ or CD8^+^ cells of ID8-*Trp53*^*−/−*^*Brca2*^−*/−*^ tumours. **h** Digital IHC analysis of intratumoral F4/80^+^ and Ki-67^+^ cells in ID8-*Trp53*^−*/−*^*Brca2*^*−/−*^Control or *Cxcl9*^*+*^ tumours. **i** Kaplan–Meier plots showing time to ascites formation or overall survival for ID8-*Trp53*^*−/−*^*Brca2*^*−/−*^Control or *Cxcl9*^*+*^ tumour-bearing-mice (at least six mice per group, data representative of two independent experiments). **j** Kaplan–Meier plots showing time to onset of ascites or overall survival under anti-PD-L1 treatment or IgG2b control, stratified by Cxcl9 overexpression in the *Brca2-*deficient ID8 model (at least four mice per group). Bars represent mean ± SEM. Each dot represents the data of one individual mouse.

Although so far, there are no clinical data available on the impact of *BRCA* mutations or homologous repair deficiency (HRD) on anti-PD-L1 monotherapy efficacy in ovarian cancer, results from the combination therapy with PARP inhibitors indicate that the benefit of adding ICB might be higher in *BRCA* wild-type tumours [28–30]. Therefore, we next tested the impact of Cxcl9 in the *BRCA*-deficient ID8-*Trp53*^*−/−*^*Brca2*^*−/−*^ model [22]. To this end, we generated ID8-*Trp53*^*−/−*^*Brca2*^*−/−*^*empty vector* and ID8-*Trp53*^−*/−*^*Brca2*^−*/−*^*Cxcl9*^*+*^ clones (termed “*Brca2*^*−/−*^Control” or “*Brca2*^*−/−*^*Cxcl9*^*+*^”, respectively) that exhibited an in vitro Cxcl9 overexpression comparable to that of the *Brca*^*WT*^ clones (Fig. 3a and Supplementary Fig. S3A; Fig. 1a and Supplementary Fig. S1A for comparison). As in the *Brca*^*WT*^ cells, in vitro cell proliferation was not affected by *Cxcl9* overexpression in *Brca2*^*−/−*^ cells (*P* = 0.273; Fig. 3b). In vivo, Cxcl9 concentration was significantly higher in the ascites of *Brca2*^*−/−*^*Cxcl9*^*+*^ tumours than in the ascites of *Brca2*^*−/−*^Control tumours (median 3.0 vs. 1.6 pg/mL; *P* = 0.033; Fig. 3c). While Cxcl10 ascites concentrations were not affected by *Cxcl9* overexpression in *Brca2*^*−/−*^ tumour-bearing mice (median 38.6 vs. 23.8 pg/mg total protein; *P* = 0.23; Fig. 3c), they were more than fivefold higher in *Brca2*^*−/−*^ than in *Brca*^*WT*^ tumour-bearing mice (median 8.2 vs. 45.5 pg/mg total protein; *P* = 0.0002; Fig. 3d). By contrast, Cxcl9 expression was not affected by the *Brca* loss-of-function (median 1.31 vs. 1.96 pg/mg total protein; *P* = 0.26; Fig. 3d). This very well reflects the concept of pathogenic *BRCA* mutations activating the cGAS-STING pathway via cytosolic accumulation of double-stranded DNA, eventually leading to increased secretion of CXCL10, whereas CXCL9 release is augmented only to a minor extent [31]. Moreover, it helps to explain the increased number of tumour-infiltrating CD3^+^ and CD8^+^ lymphocytes reported in *BRCA*-mutated human ovarian cancers compared to *BRCA* non-mutated tumours [32], which was well reproduced in our model (Fig. 3e). In addition, *Brca2*^*−/*−^ exhibited a significantly reduced number of PD-1^+^ immune cells (Fig. 3e). In contrast to *Brca2*^*WT*^ tumours (Fig. 1e), we did not observe a further increase in the number of CD3^+^ or CD8^+^ T cells upon Cxcl9 overexpression in the *Brca2*^*−/−*^ model (Fig. 3f), possibly as a result of a saturation effect by the elevated STING-dependent chemokines, e.g. Cxcl10. In contrast to *Brca*^*WT*^ tumours, no correlation was observed between Cxcl9 expression and T-cell infiltration upon *Brca2* loss-of-function (Fig. 3g), despite similar levels of Cxcl9 overexpression in both models (see above). Likewise, there was no difference for F4/80^+^ tumour-associated macrophages or Ki-67 proliferation index (Fig. 3h).

In line with the notion of an intrinsically already-inflamed tumour microenvironment of *BRCA*-mutated tumours, *Cxcl9* overexpression in *Brca2*^−*/−*^ cells did not significantly improve the onset of ascites (median 36 vs. 32 days; *P* = 0.14) or overall survival (median 48 vs. 44 days; *P* = 0.22; Fig. 3i) to the degree as was seen in the *Brca*^*WT*^ model. Similarly, Cxcl9 overexpression did not improve anti-PD-L1 therapy in the ID8-*Trp53*^−*/−*^*Brca2*^*−/*−^ model (Fig. 3j). Neither chemokine expression nor tumour-infiltrating immune subsets were affected by Cxcl9 or the anti-PD-L1 treatment in this model (Supplementary Fig. S3B–D).

### CXCL9 does not regulate PD-L1 expression in murine or human ovarian cancer cells

**Fig. 4:**
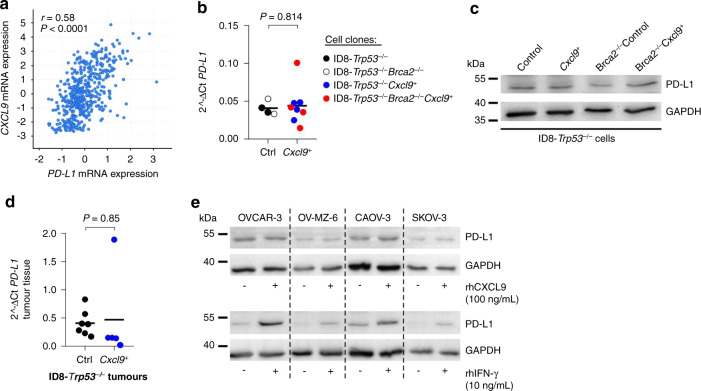
PD-L1 is not upregulated by Cxcl9 in murine or human ovarian cancer cell lines. **a** Pearson correlation analysis between *CXCL9* and *PD-L1* mRNA expression including 489 patients with ovarian serous cystadenocarcinoma from the publicly available dataset of “The Cancer Genome Atlas” (TCGA) project. **b** Quantitative PCR analysis of *PD-L1* RNA-expression normalised to Hprt housekeeping gene in ID8-*Trp53*^*−/−*^ (*n* = 2), ID8-*Trp53*^−*/−*^*Brca2*^*−/−*^ (*n* = 2), ID8-*Trp53*^*−/−*^*Cxcl9*^*+*^ (*n* = 4) and ID8-*Trp53*^*−/−*^*Brca2*^*−/−*^*Cxcl9*^*+*^ (*n* = 4) cell clones. **c** Western blot analysis of cell lysates of murine ID8-*Trp53*^−*/−*^ clones (one clone of each group presented in **b**), probed with an anti-PD-L1 antibody. **d** Quantitative PCR analysis of *PD-L1* RNA-expression normalised to Hprt housekeeping gene in mesenteric tumour tissue (ID8-*Trp53*^*−/−*^ Control (Ctrl) and ID8-*Trp53*^*−/−*^*Cxcl9*^*+*^ (*Cxcl9*^*+*^) tumours, taken from the mouse experiment shown in Fig. 1d). **e** Anti-PD-L1 western blot analysis of human ovarian cancer cell lines OVCAR-3, OV-MZ-6, CAOV-3 and SKOV-3 stimulated with recombinant human CXCL9 (100 ng/ml) or IFN-γ (10 ng/ml) for 72 h. Each dot indicates data of one cell clone or one individual mouse. Horizontal lines represent means.

Next, we set out to test the hypothesis that CXCL9 improves anti-PD-L1 treatment in *BRCA*^*WT*^ ovarian cancers due to an upregulation of PD-L1. A recent report had demonstrated this in bladder cancer cells [33], and in the publicly accessible TCGA dataset we found a moderate correlation of *CXCL9* and *PD-L1* expression, suggestive of possible interaction (*r* = 0.58 (Pearson); *P* < 0.0001; Fig. 4a). However, there was no difference in PD-L1 expression in ID8-*Trp53*^*−/−*^ or ID8-*Trp53*^*−/−*^*Brca2*^−*/−*^ cells with or without *Cxcl9* overexpression, neither on mRNA nor on protein level (Fig. 4b, c). Moreover, we did not observe a difference in *PD-L1* expression between ID8-*Trp53*^*-/-*^*Cxcl9*^*+*^ or ID8-*Trp53*^−*/−*^Control tumours in vivo (*P* = 0.851; Fig. 4d). Finally, the CXCR3-positive human ovarian cancer cell lines OVCAR-3, OV-MZ-6, CAOV-3 and SKOV-3 were treated with 100 ng/mL recombinant human CXCL9 for 72 h and lysed for western blot analysis. We did not observe an induction of PD-L1 by CXCL9 (Fig. 4e), despite CXCR3 expression and function in these cells [26]. However, as a positive control, all cells induced PD-L1 after incubation with 10 ng/mL IFN-γ (Fig. 4e).

Thus, upregulation of PD-L1 does not seem to contribute to the improvement of anti-PD-L1 therapy efficacy by CXCL9. The moderate correlation of *PD-L1* and *CXCL9* expression in human ovarian cancers most likely represents a consequence of interferon response in these tumours.

### Clear-cell carcinomas show a significantly higher CXCL9 expression compared to other histological subtypes of ovarian cancer

**Fig. 5:**
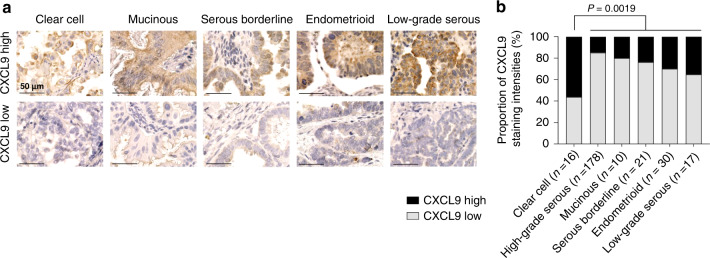
Frequency of CXCL9 overexpression is higher in clear-cell carcinomas than in other histological subtypes of ovarian cancer. **a** Representative pictures of immunohistochemical CXCL9 staining patterns in different ovarian cancer subtypes. Intensity scores +1 and +2 were summarised as “low” expression group, whereas score +3 was stated as “high” expression group (scale bar 50 µm). **b** Relative distribution of low and high CXCL9-expressing samples within each histological subtype.

We previously identified high CXCL9 expression as a strong prognostic favourable marker in high-grade serous ovarian cancer [12], which was confirmed in several genomic studies on ovarian cancer [14, 15, 19]. In addition, our preclinical findings above strongly suggest that those tumours with high CXCL9 expression display an increased response to immune checkpoint blockade. In the HGSOC subtype, the proportion of CXCL9-high tumours is rather low [12]. Clinical observations show that among the different subtypes, clear-cell ovarian cancers respond best to ICB (Table 1 and ref. [34]). To test whether this may be due to a higher proportion of CXCL9-high tumours in this histological subtype, we now evaluated CXCL9 expression in a cohort of other histological subtypes of ovarian cancer. Semiquantitative assessment of CXCL9 expression was performed as described before [12], and CXCL9 expression was dichotomised into low or high expression (Fig. 5a). The proportion of CXCL9^high^ tumours was significantly higher in ovarian clear-cell carcinomas than in all other subtypes (*P* = 0.019; Fig. 5b). More specifically, we analysed 16 clear-cell cases (56% CXCL9^high^ vs. 44% CXCL9^low^), 178 high-grade cases (15% high vs. 85% low [12]), 10 mucinous cases (20% high vs. 80% low), 21 borderline cases (24% high vs. 76% low), 30 endometrioid cases (30% high vs. 70% low) and 17 low-grade serous ovarian cancers (35% high vs. 65% low). Patient characteristics are summarised in Supplementary Table S1. Strikingly, this observation matches the clinical observation that clear-cell ovarian cancers responded best to immune checkpoint inhibition in clinical Phase II/III trials of ovarian cancer so far as well as to atezolizumab maintenance therapy after first-line chemotherapy [27].

**Table 1:** Overall response rates (ORR) and histologic subtypes in Phase II trials of anti-PD-1/PD-L1 therapy in ovarian cancer.

Trial	Monotherapy	ORR	Literature
UMIN00000571	Nivolumab	1/2 clear cell (50%) 2/18 others (11%)	Hamanishi et al., 2015 [48]
NCT01375842	Atezolizumab	1/1 clear cell (100%) 1/9 others (11%)	Liu et al., 2019 [49]
NCT02674061 (KEYNOTE-100)	Pembrolizumab	3/19 clear cell (16%) 24/332 others (7.2%)	Matulonis et al., 2019 [50] Matulonis et al., 2020 [51]
NCT01772004 (JAVELIN Solid Tumour)	Avelumab	2/2 clear cell (100%) 10/123 others (8.1%)	Disis et al., 2019 [52]

## Discussion

Response to immune checkpoint inhibitor monotherapy has remained disappointingly low in ovarian cancer, demonstrating the urgent clinical need to decipher the underlying mechanisms of resistance and to identify suitable predictive biomarkers. As mentioned above, the reasons for ICB resistance may be found in (1) a comparatively low tumour mutational burden (TMB), (2) an immune-suppressive cellular microenvironment enriched with regulatory T cells and tumour-associated macrophages, and (3) an insufficient infiltration of the tumour with effector T cells. In solid tumours, CXCL9, along with the other CXCR3 chemokines, has been made responsible for this tumour-suppressive lymphocytic infiltration. In human ovarian cancer, the CXCR3 receptor is overrepresented on tumour-infiltrating T cells compared with peripheral lymphocytes, highlighting its importance in immune infiltration [13]. Consistently, several studies identified upregulation of these chemokine genes as characteristic of the so-called ‚immunoreactive subtype‘, that is associated with better response to ICB and with the best prognosis of all high-grade serous ovarian cancer subtypes. In this study, we demonstrate that CXCL9 is not only a surrogate for an enhanced, interferon-driven immune response, but that its overexpression improves immune infiltration, thereby inhibiting tumour growth. This finding functionally supports our description of CXCL9 protein expression as a robust and independent prognostic marker in human high-grade serous ovarian cancer [12]. Expanding prior reports already suggesting involvement of CXCR3 chemokines in the ICB mechanism of action, our study is, to the best of our knowledge, the first to demonstrate that CXCL9 is all by itself sufficient to overcome ICB resistance in ovarian cancer. Based on our findings, we postulate that this is due to an enhanced immune infiltration and/or activation, and not to a PD-L1 upregulation that has been attributed to CXCR3 chemokines in the cancer microenvironment before [33, 35]. However, a limitation of our experimental approach is that we neglect other immune cell cytokine influences that normally affect CXCL9 expression in tumour cells. On the other hand, it allows us to directly attribute observed effects to CXCL9 overexpression within the tumour microenvironment.

In its capacity as a driver of ICB response, CXCL9 might as well be a predictive biomarker to identify the hitherto small number of ovarian cancer patients that showed durable responses under ICB monotherapy, as PD-L1 expression was shown not to predict therapy response satisfactorily. This concept has already been confirmed across various entities such as non-small-cell lung cancer or melanoma, in which *CXCL9* gene expression emerged, besides TMB, as the strongest predictor of ICB response [11, 36–38]. Extending this approach to ovarian cancer, retrospectively testing the predictive power of CXCL9 expression in prospectively conducted clinical trials could be an easy-to-perform next step, e.g. in the IMagyn050 trial which tested the benefit of additional anti-PD-L1 maintenance therapy after first-line chemotherapy in ovarian cancer [27]. Interestingly, we found the highest number of CXCL9-overexpressing tumours in the clear-cell subtype, which is the subtype that responded best in the ICB monotherapy trials so far [3]. Moreover, a recent study by Terzic et al. might further corroborate our rationale of Cxcl9 driving ICB since it reported a sustained response to first-line pembrolizumab monotherapy in a case of *CSMD3*-mutated high-grade serous ovarian cancer that is characterised by a strong upregulation of *CXCL9* [39].

In order to therapeutically exploit our results, ICB should be combined with therapies that induce the expression of CXCR3 chemokines in the ovarian cancer tumour microenvironment. Prominent examples include CDK4/6 inhibitors such as abemaciclib [40], epigenetic modulators [41] or PARP inhibitors (PARPi) [42]. The latter ones have already been combined with ICB in several clinical Phase II trials of ovarian cancer, achieving encouraging response rates of more than 50% in pretreated, partly platinum-resistant tumours [28–30, 34]. This synergistic effect has been, inter alia, attributed to the PARP inhibitor-induced activation of the STING pathway and the subsequent release of CXCR3 ligands [31]. Although it has been shown that a successful PARPi/ICB combination depends on the adaptive immune system, the indispensable role of the CXCR3 chemokine system therein has yet to be proven [43]. However, our in vivo results of CXCL9 promoting ICB efficacy in ovarian cancer fuel this assumption. Moreover, in a Phase II trial conducted by Lampert and colleagues, testing the combination of the PARP inhibitor olaparib and the anti-PD-L1 antibody durvalumab, sequentially obtained tumour biopsies revealed an upregulation of CXCL9 and an induction of TILs 14 days after onset of therapy [20]. This interferon response as well as the immunoreactive subtype—and not TMB—were deemed predictive for the response. It remains to be determined if PARPi or ICB (or both) caused this increase in chemokine expression. In our study, we did not observe an increase in Cxcl9 upon anti-PD-L1 treatment in the ascites of mice, suggesting a more prominent role for PARPi in this matter. However, in other preclinical models, CXCR3 ligands were upregulated after immune checkpoint inhibition [9]. While PARP inhibition directly induces CXCR3 chemokines via the STING pathway, immune checkpoint inhibitors might do so indirectly through the creation of an interferon-enriched environment.

In the same way as PARP inhibitors, *BRCA* loss-of-function mutations can cause a cytosolic accumulation of DNA fragments, eventually activating the STING pathway and leading to chemokine release [44]. Our results confirm this paradigm now in preclinical ovarian cancer. While we did observe only a slight, non-significant increase in Cxcl9 ascites concentration upon *Brca2* loss, Cxcl10 was more than fivefold higher than in *Brca2*^*WT*^ tumours. This was accompanied by a doubling of tumour-infiltrating CD3^+^ or CD8^+^ T cells. Although CXCR3 chemokine expression has not yet been compared as a function of *BRCA* mutation status in human ovarian cancer, our observation is sufficient to explain the higher number of tumour-infiltrating lymphocytes in *BRCA-*mutated tumours [32]. However, we could not confirm the higher expression of PD-1 and PD-L1 upon *BRCA* loss-of-function reported in human ovarian cancer [32, 45]. In fact, in murine ovarian cancer, PD-L1 expression was unchanged in vitro and in vivo, while the number of PD-1-positive cells was even decreased in *Brca2*^*−/−*^ tumours. The already-inflamed baseline state of *Brca2*^−*/−*^ tumours (Cxcl10^high^, more T cells) and the reduced expression of PD-1 both help to explain why similar expression levels of Cxcl9 as in the *Brca2*^*WT*^ tumours did not cause a significant survival benefit in the *Brca2*^*−/−*^ tumours. Moreover, the beneficial effects could not further be improved by anti-PD-L1 therapy in our study. Although data on the efficacy of ICB monotherapy in ovarian cancer based on *BRCA* mutation status is still sparse [46], our findings reflect the results from Phase II trials on the ICB/PARPi combination therapy. In the MEDIOLA trial, for instance, PARPi (olaparib) and ICB (durvalumab) showed a synergism only in the *BRCA* non-mutated cohort [29, 30], while the impressive response rate of 72% in the *BRCA*-mutated cohort was numerically the same as in the SOLO-3 trial, testing olaparib alone in a very similar patient cohort (with all due caution concerning cross-trial comparisons) [47]. Thus, ICB did not work in these already-inflamed tumours. However, results from the ongoing Phase III trials have to be awaited.

In conclusion, our study provides a functional basis for the protective nature of the CXCL9 chemokine and identifies it as a sufficient driver of successful immune checkpoint blockade in ovarian cancer. However, this impact dispersed in the *BRCA*-deficient ovarian cancer model, most likely as a result of an environment already enriched with CXCR3 ligands. The capacity to induce CXCL9 in the tumour microenvironment should thus be an eligibility criterion to efficiently select ICB therapy partners in the future. Moreover, CXCL9 might serve as a reasonable biomarker to predict ICB response in patients suffering from this deadly disease.

## Supplementary information

Reproducibility checklist

Supplementary Material and Methods and Figures

Table S1

## Data Availability

The other data generated or analysed are included in this article or available from the corresponding author upon reasonable request.
