# Supplementary material for: CXCL9 inhibits tumour growth and drives anti-PD-L1 therapy in ovarian cancer
Source: Br J Cancer. 2022 Mar 21;126(10):1470–80. doi: 10.1038/s41416-022-01763-0 (PMC9090786; doi:10.1038/s41416-022-01763-0)
Supplement: Supplementary file 2 — Supplementary Material and Methods and Figures [file 41416_2022_1763_MOESM2_ESM.pdf]

Supplementary Material and Methods

Quantitative reverse transcriptase PCR. For relative gene expression analysis, RNA was extracted from cell pellets or granulated tumor tissue using the RNeasy® Plus Mini Kit (Cat. #74134) from Qiagen following the manufacturer's protocol. 1 µg of total RNA was used to synthesize cDNA via SuperScript™ IV First-Strand Synthesis System (Cat. #18091050) from Thermo Fisher Scientific. cDNA (150 ng) was amplified using Brilliant III Ultra-Fast QPCR Master Mix (Cat. #600890-51, Agilent Technologies), universal probes for murine Cxcl9 (Probe #1 Cat. #318501) and murine Hprt (Probe #95 Cat. #263935, Roche Molecular Systems) as well as corresponding primer pairs targeting the full-length proteins (mCxcl9 forward: 5'-GCA TCG ATT CCT TAT CA-3'; mCxcl9 reverse: 5'-CTT TTC CTC TTG GGC ATC AT-3'; mHprt forward: 5'-CCT CCT CAG ACC GCT TTT T-3'; mHrprt reverse: 5'-AAC CTG GTT CAT CAT CGC TAA-3'; Metabion). For quantification of PD-L1 and Hprt, Thermo Fisher probes supplemented with associated primers were used (#Mm00452054_m1 Cd274, #Mm03024075_m1 Hprt, Thermo Fisher Scientific). Cycle parameters were as follows: 3 minutes: 95°C; 40X (15 s: 95 °C; 1 minute: 60°C). Gene expression levels were normalized to Hprt housekeeping gene and quantification was performed using the ΔCt calculation method.

MTT proliferation assay. Metabolic activity was compared by MTT assay. Briefly, cells were seeded at $2x10^3$ per well in 96-well plate triplicates within 70 µl cell culture media (DMEM with 5% FCS). After 4, 24 and 48 hours, 14 µl MTT reagent was added for 3 h at 37°C. Subsequently, cells were lysed by adding 150 µl DMSO and optical density was measured at 570 nm in a microplate reader.

Western Blot analysis. Murine cells were collected as cell pellets without treatment. Human cells were seeded at 50% confluency overnight in 6-well plates and treated with 100 ng/mL recombinant human CXCL9 (Cat. #300-26, Peprotech) or 10 ng/mL human IFNγ (Cat. #300-02, Peprotech) for 72 h. Protein concentrations of cell lysates were assessed using Pierce™

BCA Protein Assay Kit (Cat. #23225, Thermo Fisher Scientific) according to the manufacturer's instructions. 30 µg of cell lysates were separated on 12% SDS-polyacrylamide gels and electrotransferred to PVDF membranes using standard procedures. Membranes were blocked with 5% skim milk in TBS-Tween20 (0.05%) for 1 hour at room temperature. Primary antibody incubation was conducted overnight at 4°C with 1:600 polyclonal rabbit anti-PD-L1/CD274 (Cat. #17952-1-AP, Proteintech). Corresponding secondary goat-anti-rabbit IgG-HRP antibody (1:10000, Cat. #G21234, Thermo Fisher Scientific) was incubated for 1 hour at room temperature. Between incubation steps, membranes were washed 3 x 10 min with TBS-T. Blots were developed using Immobilon® Western Chemiluminescent HRP Substrate (Cat. #WBKLS0500, Millipore) and signals were detected using Bio-Rad ChemiDoc™ XRS+. GAPDH was used as a loading control (1:10000 monoclonal mouse anti GAPDH, Cat. #MAB374, Sigma-Aldrich and 1:10000 goat-anti-mouse IgG-HRP, Cat. #G21040, Invitrogen).

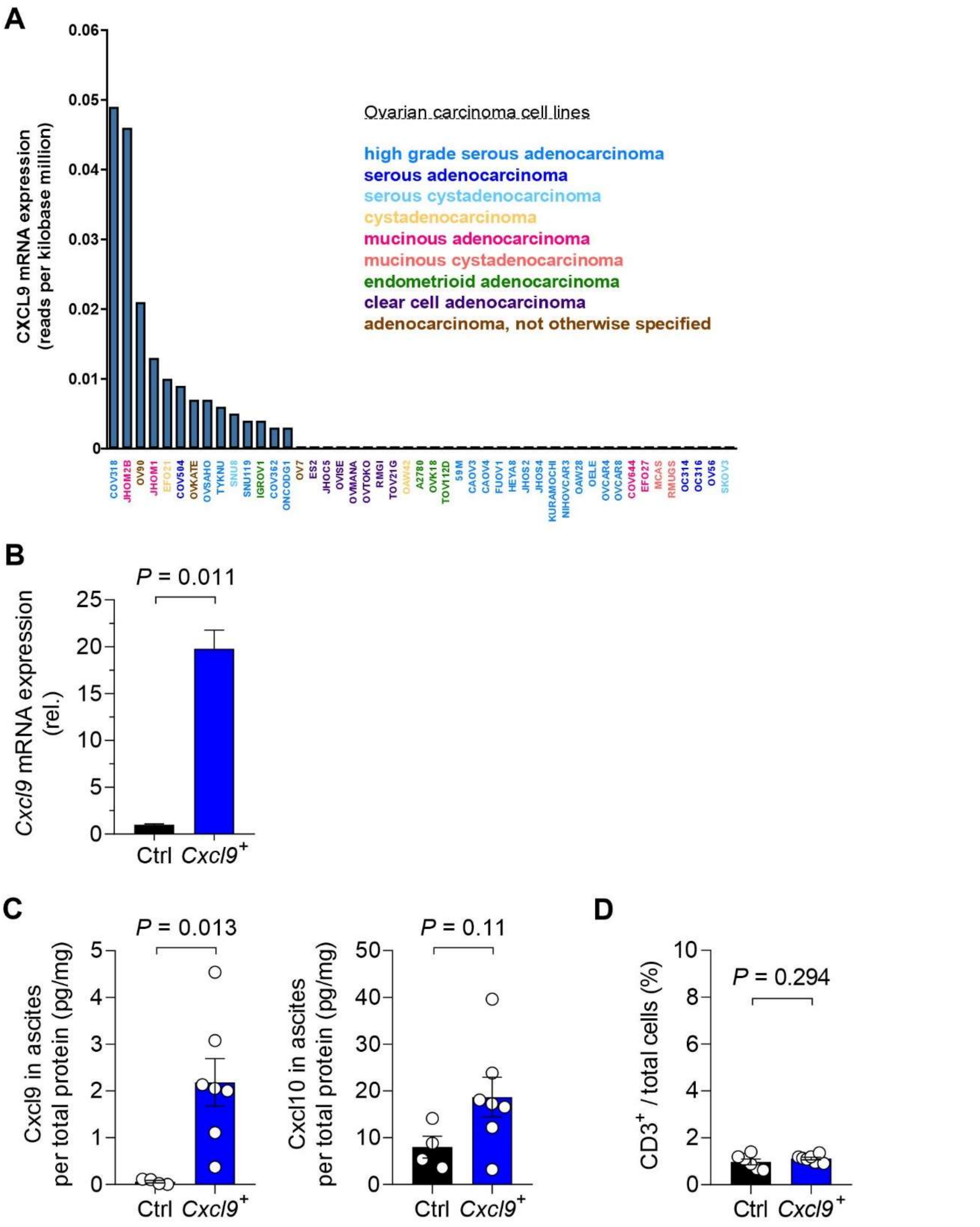

Figure S1. A, *CXCL9* mRNA expression values given as “RPKM” (reads per kilobase million) in 47 different ovarian carcinoma cell lines including 8 distinct histological subtypes (publicly available data set was obtained from cBioPortal website). **B,** Verification of *Cxcl9* overexpression at RNA-level via quantitative PCR analysis of ID8-*Trp53*$^{-/-}$Control (Ctrl) and ID8-*Trp53*$^{-/-}$*Cxcl9*$^{+}$ (*Cxcl9*$^{+}$) cells. **C,** Soluble Cxcl9 or Cxcl10 measured in ascites of athymic nude mice inoculated with ID8-*Trp53*$^{-/-}$Control or *Cxcl9*$^{+}$ cells via ELISA and normalized to total

protein amount. **D,** Digital Analysis of intratumoral $CD3^+$ T-cells within ID8-*Trp53*$^{-/-}$Control or *Cxcl9*$^+$ tumors in the athymic nude model. Bars represent mean ± SEM. Each dot indicates data of one individual mouse.

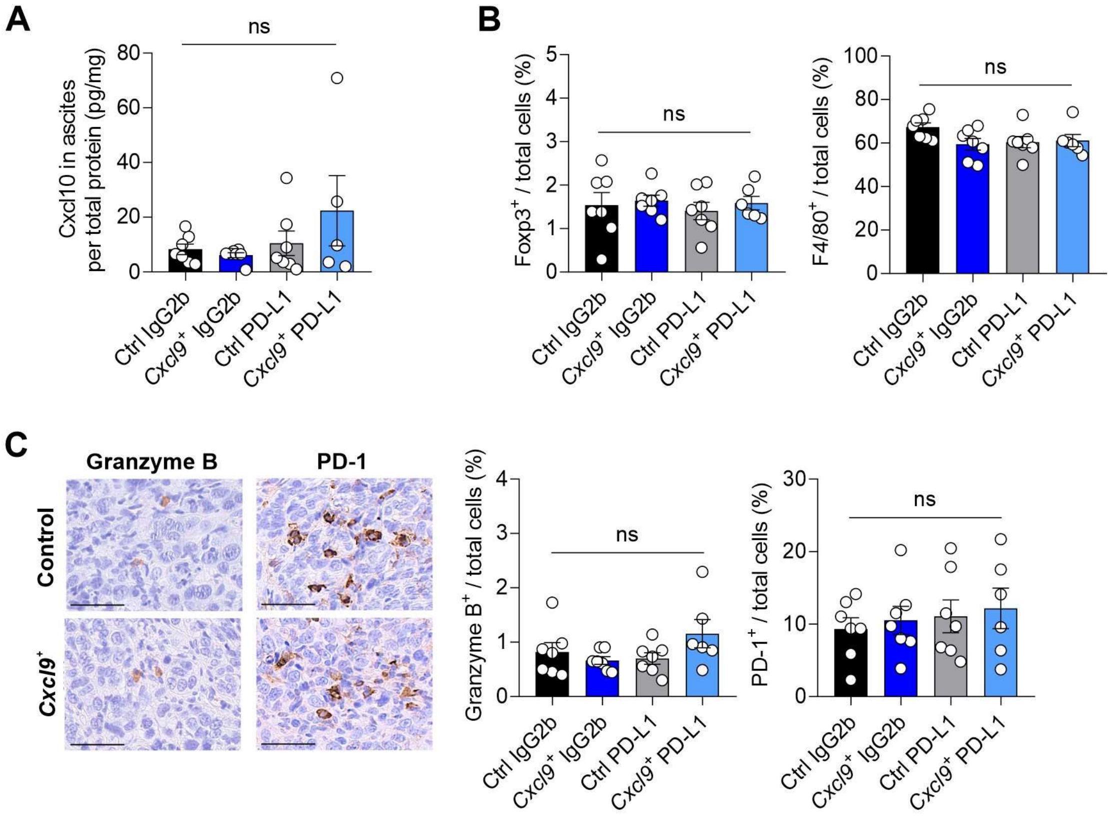

Figure S2. A, Soluble Cxcl10 measured via ELISA in ascites of anti-PD-L1 or IgG2b treated mice stratified by Cxcl9 overexpression in the ID8-*Trp53*$^{-/-}$ model. Values were normalized to total protein amount. **B-C,** Digital analysis of immunohistochemically detected immune cell population markers (Foxp3, F4/80, Granzyme B, PD-1). Representative IHC pictures of Granzyme B and PD-1 staining are presented with 50 µm scale bars. Each dot in A-C indicates data of one individual mouse. Bars represent mean ± SEM.

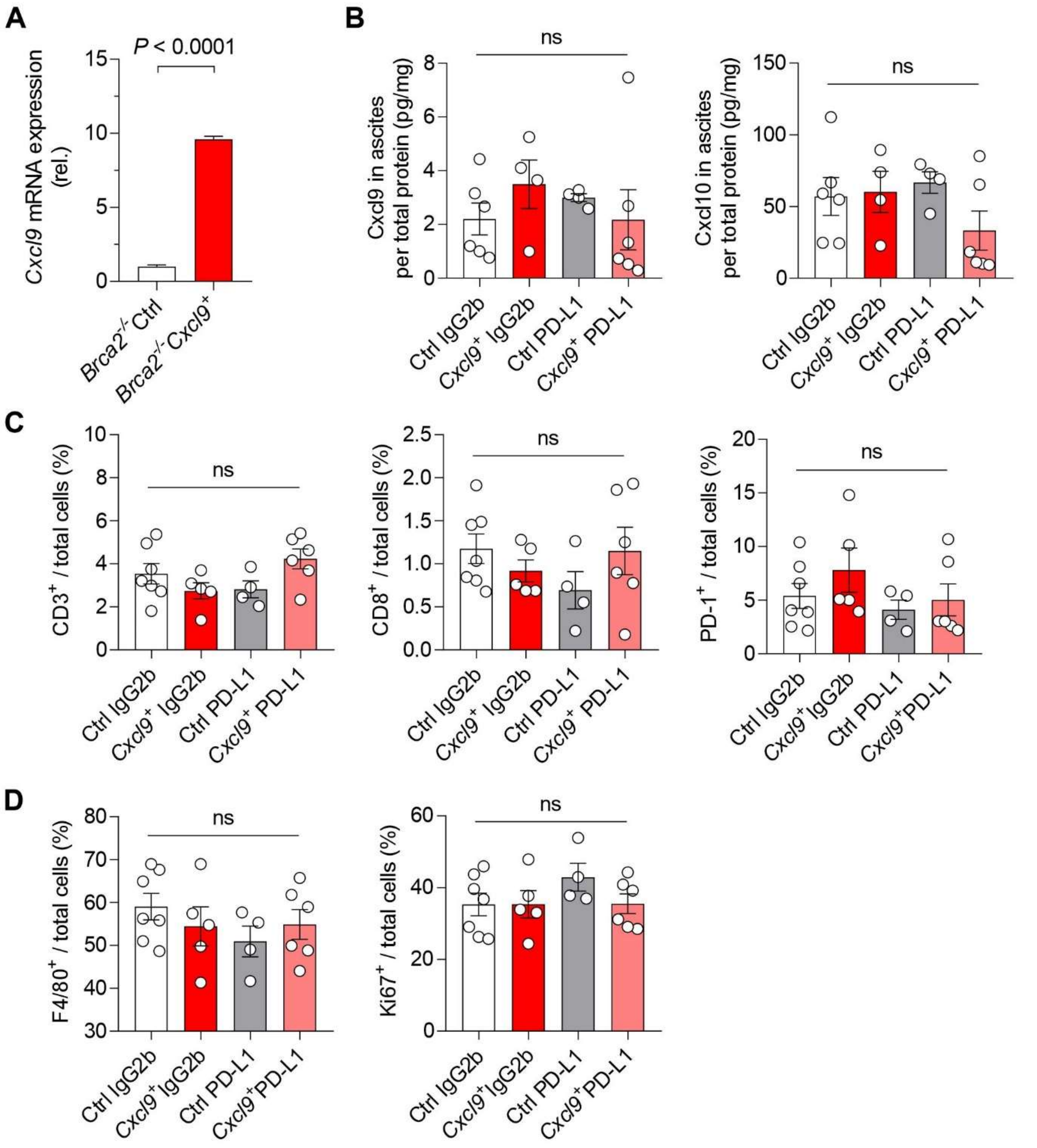

Figure S3. A, Lentiviral overexpression of Cxcl9 in ID8-*Trp53*$^{-/-}$*Brca2*$^{-/-}$ cells (*Brca2*$^{-/-}$*Cxcl9*$^{+}$) was tested via quantitative PCR and compared to ID8-*Trp53*$^{-/-}$*Brca2*$^{-/-}$ empty vector (*Brca2*$^{-/-}$ Ctrl) cells. **B,** Soluble Cxcl9 or Cxcl10 in ascites samples of anti-PD-L1 or IgG2b treated mice stratified by Cxcl9 overexpression in the *Brca2*-deficient model was quantified by ELISA and normalized to total protein amount. **C-D,** Digital analysis of immunohistochemical markers (CD3, CD8, PD-1, F4/80, Ki-67) within the tumor microenvironment. Bars represent mean ± SEM. Each dot indicates data of one individual mouse.
